# Disease-related determinants are associated with mortality in dementia due to Alzheimer’s disease

**DOI:** 10.1186/s13195-018-0348-0

**Published:** 2018-02-20

**Authors:** Hanneke F. M. Rhodius-Meester, Hilkka Liedes, Ted Koene, Afina W. Lemstra, Charlotte E. Teunissen, Frederik Barkhof, Philip Scheltens, Mark van Gils, Jyrki Lötjönen, Wiesje M. van der Flier

**Affiliations:** 1 Alzheimer Center, Department of Neurology, VU University Medical Center, Amsterdam Neuroscience, P.O. Box 7057, 1007 MB Amsterdam, The Netherlands; 2 VTT Technical Research Center of Finland Ltd, Tampere, Finland; 3 Department of Medical Psychology, VU University Medical Center, Amsterdam Neuroscience, Amsterdam, The Netherlands; 4 Neurochemistry Lab and Biobank, Department of Clinical Chemistry, VU University Medical Center, Amsterdam Neuroscience, Amsterdam, The Netherlands; 5 Department of Radiology and Nuclear Medicine, VU University Medical Center, Amsterdam Neuroscience, Amsterdam, The Netherlands; 6 Institute of Neurology, UCL, London, UK; 7 Institute of Healthcare Engineering, UCL, London, UK; 8 Combinostics Ltd, Tampere, Finland; 9 Department of Epidemiology and Biostatistics, VU University Medical Center, Amsterdam Neuroscience, Amsterdam, The Netherlands

**Keywords:** Alzheimer’s disease, Prognosis, Mortality, Diagnostic test assessment

## Abstract

**Background:**

Survival after dementia diagnosis varies considerably. Previous studies were focused mainly on factors related to demographics and comorbidity rather than on Alzheimer’s disease (AD)-related determinants. We set out to answer the question whether markers with proven diagnostic value also have prognostic value. We aimed to identify disease-related determinants associated with mortality in patients with AD.

**Methods:**

We included 616 patients (50% female; age 67 ± 8 years; mean Mini Mental State Examination score 22 ± 3) with dementia due to AD from the Amsterdam Dementia Cohort. Information on mortality was obtained from the Dutch Municipal Register. We used age- and sex-adjusted Cox proportional hazards analysis to study associations of baseline demographics, comorbidity, neuropsychology, magnetic resonance imaging (MRI) (medial temporal lobe, global cortical and parietal atrophy, and measures of small vessel disease), and cerebrospinal fluid (CSF) (β-amyloid 1–42, total tau, and tau phosphorylated at threonine 181 [p-tau]) with mortality (outcome). In addition, we built a multivariate model using forward selection.

**Results:**

After an average of 4.9 ± 2.0 years, 213 (35%) patients had died. Age- and sex-adjusted Cox models showed that older age (HR 1.29 [95% CI 1.12–1.48]), male sex (HR 1.60 [95% CI 1.22–2.11]), worse scores on cognitive functioning (HR 1.14 [95% CI 1.01-1.30] to 1.31 [95% CI 1.13–1.52]), and more global and hippocampal atrophy on MRI (HR 1.18 [95% CI 1.01-1.37] and HR 1.18 [95% CI 1.02-1.37]) were associated with increased risk of mortality. There were no associations with comorbidity, level of activities of daily living, apolipoprotein E (*APOE*) ε4 status, or duration of disease. Using forward selection, the multivariate model included a panel of age, sex, cognitive tests, atrophy of the medial temporal lobe, and CSF p-tau.

**Conclusions:**

In this relatively young sample of patients with AD, disease-related determinants were associated with an increased risk of mortality, whereas neither comorbidity nor *APOE* genotype had any prognostic value.

**Electronic supplementary material:**

The online version of this article (10.1186/s13195-018-0348-0) contains supplementary material, which is available to authorized users.

## Background

Dementia due to Alzheimer’s disease (AD) is, by definition, a progressive disorder [1]. For patients, a diagnosis is not the endpoint, but rather the beginning of the subsequent trajectory of disease. Physicians are fairly good at establishing an accurate diagnosis, but they are hardly able to predict the course of the disease for the individual patient. In general, patients with dementia due to AD have a shorter life expectancy than the general population, with average survival being between 5 and 10 years [2–4]. Yet, survival time varies considerably between individuals.

Determinants of mortality in AD have been examined in various studies. Most have been focused on demographic factors or on clinical factors such as severity of cognitive impairment, dependency, and comorbidity [5–9]. Male sex and older age have been associated with increased risk of mortality in AD [2, 3, 10]. Cardiovascular diseases and risk factors such as hypertension and diabetes mellitus have been identified as determinants of mortality, but only in studies of older patients with dementia [11–15]. Few studies have been focused on AD-specific factors, such as cerebrospinal fluid (CSF) and magnetic resonance imaging (MRI) markers. More severe neuronal degeneration, as reflected by a high total tau (tau) concentration and whole-brain atrophy, has been suggested as a determinant of mortality [12, 16–18]. In one study, microbleeds were associated with mortality in AD, and white matter hyperintensities (WMH) were associated with mortality in all-cause dementia [12].

Researchers in previous studies tended to evaluate only a few prognostic factors per study and included mainly patients aged 75 years and older, who are at risk of mortality owing to their advanced age even without a dementia diagnosis [3, 19]. Prognostic factors may be different for patients with early-onset AD, who are younger and have less comorbidity but are prone to a more aggressive disease course [10, 20–22]. We aimed to investigate the prognostic value of baseline clinical data, including demographics, comorbidity, neuropsychology, and CSF and MRI biomarkers, as determinants of mortality in dementia due to AD.

## Methods

### Patients

In this longitudinal study, we included 616 patients with a baseline diagnosis of dementia due to AD from a memory clinic-based cohort (the Amsterdam Dementia Cohort) who had a baseline visit between 2000 and 2014 [23]. Subjects were selected if a neuropsychological test battery was available at baseline, with a baseline Mini Mental State Examination (MMSE) score ≥ 16 and a minimum follow-up of 2 years. At baseline, patients received a standardized and multidisciplinary workup, including medical history; physical, neurological, and neuropsychological examinations; MRI; laboratory tests; and lumbar puncture for CSF measurements. Years of education and self-reported duration of complaints were recorded. For the assessment of activities of daily living, we used the Disability Assessment for Dementia (DAD) [24]. We included all data that were collected within 6 months of baseline diagnosis. Diagnoses were made in a multidisciplinary consensus meeting. Patients were diagnosed with probable AD using the criteria of the National Institute of Neurological and Communicative Disorders and Stroke/Alzheimer’s Disease and Related Disorders Association; all patients also met the core clinical criteria of the National Institute on Aging-Alzheimer’s Association for AD dementia [25, 26].

### Medical history

We recorded and defined the presence (yes/no) of hypertension (history of hypertension and/or use of antihypertensive drugs), hypercholesterolemia (history of hypercholesterolemia and/or use of cholesterol-lowering drugs), diabetes (history of diabetes mellitus and/or use of antidiabetic drugs), and cardiovascular disease (at least one of the following: history of coronary heart disease, heart failure, heart disease, peripheral vascular disease, stroke, and/or transient ischemic attack). Furthermore, we dichotomized smoking status (never smoked versus current or history of smoking) and counted the medications used per patient.

### Neuropsychological tests

Cognitive function was assessed at baseline with a standardized test battery in which the MMSE was used for global cognitive functioning [27]. For memory, the Visual Association Test (VAT) and the Rey Auditory Verbal Learning Task (RAVLT) were included [28, 29]. To measure mental speed and attention, we used Trail Making Test A (TMT-A) and the forward condition of the digit span. Trail Making Test B (TMT-B) and the backward condition of the digit span were used for executive functioning [30, 31]. Language and executive functioning were tested by category fluency (animals) [32]. Missing data ranged from *n* = 19 (3%) (digit span forward) to *n* = 67 (11%) (RAVLT, delayed recall).

### MRI

Subjects were scanned as part of clinical workup using a standardized protocol on a 1.0-, 1.5-, or 3.0-T system. All scans were visually rated by trained raters and subsequently evaluated in a consensus meeting with an experienced neuroradiologist [23]. Visual rating of medial temporal lobe atrophy (MTA) was performed using coronal T1-weighted images on a 5-point (0–4) scale from the average score of the left and right sides [33]. Global cortical atrophy (GCA) was assessed visually on axial fluid-attenuated inversion recovery (FLAIR) images (range of scores 0–3) [34]. Parietal atrophy was rated using T1-weighted and FLAIR weighted images viewed in sagittal, axial, and coronal planes by computing an average score of the left and right sides (range 0–3) [35]. WMH were rated on axial FLAIR images using a four-step scale (range 0–3) [36]. Lacunes were defined as deep lesions (3–15 mm) with CSF-like signals on all sequences and were dichotomized as present or absent. Microbleeds were defined as small, round foci of hypointense signal up to 10 mm in brain parenchyma on T2*-weighted gradient echo images. The total number of microbleeds was counted and divided into three categories: zero, one or two, and three or more microbleeds. MRI data were available for 485 (79%) subjects.

### CSF

CSF analyses were performed at the Neurochemistry Laboratory at the Department of Clinical Chemistry of the VUmc. CSF was obtained by lumbar puncture between the L3-L4 or L4-L5 intervertebral space by using a 25-gauge needle and collected into polypropylene tubes. Within 2 h, the CSF was centrifuged at 1800 × *g* for 10 minutes at 4 °C, transferred to new polypropylene tubes, and stored at −20 °C until biomarker analysis (within 2 months). β-Amyloid 1–42 (Aβ_42_), tau, and tau phosphorylated at threonine 181 (p-tau) were measured with commercially available enzyme-linked immunosorbent assays (Innotest; Fujirebio, Ghent, Belgium) [37]. CSF data were available for 466 (76%) subjects.

### *APOE* genotyping

DNA was isolated from 10 ml of ethylenediaminetetraacetic acid blood. Apolipoprotein E (*APOE*) genotype was determined using the LightCycler *APOE* mutation detection method (Roche Diagnostics GmbH, Mannheim, Germany). According to *APOE* ε4 allele status, patients were dichotomized into carriers (hetero- and homozygous) and noncarriers. *APOE* status was available for 562 (91%) subjects.

### Outcome measure

For each patient, we obtained information on all-cause mortality (died yes/no with a date of death) from the Dutch municipal population register. This register was searched on 19 October 2016. Causes of death cannot be determined from this municipal registry. We defined follow-up duration as the time between the date of baseline AD diagnosis and the date of death or, if alive, between the date of baseline AD diagnosis and 19 October 2016.

### Statistical analyses

Statistical analyses were performed using IBM SPSS Statistics version 22 software (IBM, Armonk, NY, USA). *p* < 0.05 was considered significant. Baseline characteristics were compared using parametric and nonparametric tests when appropriate. We used pattern analysis to explore the amount and randomness of missing data. Because missing data were at random, but not completely at random, we imputed all missing data imputed using multiple imputation, in which the missing values were estimated on the basis of other available baseline variables in 15 imputation cycles.

To allow comparison of results on different tests within patients, all continuous variables were standardized to z-scores. All neuropsychological tests, except TMT-A and TMT-B, as well as CSF Aβ_42_, were inverted by computing −1 × z-score, with the result being that a higher score implied more advanced disease. We used Cox proportional hazards models to assess associations between all baseline determinants and mortality, taking into account time to death, using the pooled results of the 15 imputations. Each measure was assessed unadjusted (model 1), adjusted for age and sex (model 2), and adjusted additionally for MMSE and duration of complaints as a proxy of disease severity (model 3). Effect modification, using interaction terms for each variable with *age and *sex, was not found. Subsequently, we aimed to select the optimal combination of determinants by constructing a multivariate model using forward selection. The model was built by assessing all variables and consecutively selecting the variable with the lowest *p* value in a stepwise manner until *p* was < 0.10. In case of several variables with the same lowest *p* value, we calculated the Wald statistics and selected the variable with the highest Wald value. Variables were added only when the overall model improved, as evaluated using the −2 log-likelihood ratio. In an additional set of analyses, we performed similar analyses based on nonimputed data, and the results were comparable (*see* Additional file 1: Table S1 and S2). Finally, we created Kaplan-Meier curves for each of the variables selected by forward selection. Because all variables except for sex were continuous values, we used tertiles for the survival curves. Data are represented as HRs with accompanying 95% CIs.

## Results

Table 1 presents the baseline characteristics of the patients. After a follow-up of 4.9 ± 2.0 years, 213(35%) patients had died (duration baseline AD diagnosis to death 4.3 ± 2.1 years) and 403(65%) patients were alive (follow-up duration 5.3 ± 1.8 years) on the 19^th^ October 2016. Patients who had died were more often male, older and more often had cardiovascular disease. There was no difference in self-reported duration of complaints or years or activities of daily living (as measured with the DAD.

**Table 1:** Baseline characteristics of patients with Alzheimer’s disease according to outcome

	No. of patients	Alive (*n* = 403)	Died (*n* = 213)	*p* Value
Demographics				
Female sex, *n* (%)	616	218 (54)	91 (43)	0.007
Age, years	616	66 ± 7	69 ± 9	0.000
*APOE* ε4 carrier, *n* (%)	562	250 (67)	119 (63)	0.280
Years of education	616	11 ± 3	11 ± 3	0.675
Years of complaints	611	3.2 ± 2.6	2.8 ± 2.0	0.066
Years to outcome	616	5.3 ± 1.8	4.3 ± 2.1	0.000
Activities of daily living (DAD)	372	83 ± 17	82 ± 17	0.450
Medical history				
Smoking, *n* (%)	599	185 (47)	98 (49)	0.640
Hypertension, *n* (%)	616	127 (32)	77 (36)	0.245
Hypercholesterolemia, *n* (%)	616	103 (26)	46 (22)	0.275
Diabetes mellitus, *n* (%)	616	31 (8)	15 (7)	0.770
Cardiovascular disease, *n* (%)	616	71 (18)	53 (25)	0.032
No. of medications	616	2.0 ± 2.0	2.4 ± 2.1	0.062

*Abbreviations:* *APOE* Apolipoprotein E, *DAD* Disability Assessment for Dementia (range 0–100)

Years to outcome: in case of alive, follow-up duration; in case of died, duration to death

Data are presented as mean ± SD unless otherwise specified. Group differences were calculated using Student’s *t* test for continuous variables. For categorical variables, the chi-square test was used

Patients who had died performed worse at baseline on TMT-A and RAVLT immediate recall, but MMSE scores and performance on the other cognitive tests were similar. In addition, these patients’ biomarkers were indicative of more severe AD pathology, with a higher MTA and GCA, lower Aβ_42_, and higher p-tau values (Table 2).

**Table 2:** Disease-specific characteristics at baseline, according to outcome

	No. of patients	Alive (*n* = 403)	Died (*n* = 213)	*p* Value
Cognitive tests				
MMSE	616	22 ± 3	22 ± 3	0.480
Digit span forward	597	11 ± 3	11 ± 3	0.908
Digit span backward	593	7 ± 3	6 ± 2	0.154
VAT naming	576	11 ± 1	11 ± 2	0.194
VAT memory	579	6 ± 4	6 ± 4	0.641
TMT-A, seconds	581	81 ± 62	92 ± 64	0.046
TMT-B, seconds	581	299 ± 235	329 ± 215	0.079
RAVLT, immediate recall	551	23 ± 7	22 ± 8	0.026
RAVLT, delayed recall	549	2 ± 2	2 ± 2	0.391
Category fluency	563	13 ± 5	13 ± 6	0.325
MRI				
MTA	484	1.2 ± 0.8	1.6 ± 0.9	0.000
PA	470	1.2 ± 0.8	1.3 ± 0.8	0.185
GCA	482	1.0 ± 0.6	1.2 ± 0.7	0.004
WMH	485	1.0 ± 0.8	1.1 ± 0.9	0.152
Lacunes present, *n* (%)	483	20 (6)	16 (9)	0.262
Microbleeds by category, *n* (%)	393			0.064
0 microbleeds		181 (75)	112 (74)	
1–2 microbleeds		41 (17)	17 (11)	
≥ 3 microbleeds		20 (8)	22 (15)	
Infarcts present, n (%)	482	3 (1)	4 (2)	0.257
CSF				
Aβ_42_, pg/ml	466	525 ± 172	490 ± 173	0.037
tau, pg/ml	460	662 ± 340	695 ± 434	0.374
p-tau, pg/ml	463	83 ± 33	91 ± 45	0.031

*Abbreviations: MMSE* Mini Mental State Examination (score range 0–30), *Digit span forward and backward* (range 0–21), *VAT* Visual Association Test (naming range 0–12, memory range 0–12), *TMT* Trail Making Test (no range), *RAVLT* Rey Auditory Verbal Learning Task (immediate recall range 0–60, delayed recall range 0–15), *MTA* Medial temporal lobe atrophy (range 0–4; average score of left and right sides), *PA* Parietal atrophy (range 0–3; average score of left and right sides), *GCA* Global cortical atrophy (range 0–3), *WMH* White matter hyperintensities (range 0–3), *Aβ*_*42*_ β-Amyloid 1–42, *p-tau* Tau phosphorylated at threonine 181, *MRI* Magnetic resonance imaging, *CSF* Cerebrospinal fluid

Data are presented as mean ± SD unless otherwise specified. Group differences were calculated using Student’s *t* test for continuous variables. For categorical variables, the chi-square test was used

We used Cox proportional hazards models to evaluate associations between the individual determinants and mortality, taking into account time to death (Tables 3 and 4). Male sex and older age were associated with an increased risk of mortality. After adjustment for age and sex, worse performance on MMSE, digit span backward, VAT naming, TMT-A, TMT-B, and RAVLT immediate recall and category fluency were associated with an increased risk of mortality. In addition, more severe MTA and GCA seen on MRI scans were associated with an increased risk of mortality. Duration of complaints, activities of daily living (as measured with the DAD), years of education, *APOE* ε4 presence, comorbidity, MRI measures of small vessel disease, and CSF biomarkers were not associated with mortality. When we adjusted additionally for MMSE and duration of complaints as a proxy for disease severity, all related variables from model 2, except MTA, remained associated with mortality.

**Table 3:** Cox proportional hazards models used to evaluate influence of baseline characteristics and medical history on survival

	Model 1 unadjusted		Model 2 adjusted for age and sex		Model 3: model 2 plus MMSE and duration of complaints	
	HR (95% CI)	*p* value	HR (95% CI)	*p* Value	HR (95% CI)	*p* Value
Demographics						
Male sex^a^	1.57 (1.20–2.07)	0.001	1.60 (1.22–2.11)	0.001	1.79 (1.35–2.37)	0.000
Age	1.27 (1.11–1.46)	0.001	1.29 (1.12–1.48)	0.000	1.33 (1.15–1.53)	0.000
Years of education	0.99 (0.86–1.13)	0.844	0.97 (0.84–1.11)	0.636	1.0 (0.90–11.9)	0.671
Years of complaints	0.88 (0.76–1.03)	0.107	0.88 (0.76–1.03)	0.103	0.87 (0.74–1.01)	0.060
*APOE* ε4 carrier^a^	0.79 (0.59–1.06)	0.114	0.81 (0.61–1.09)	0.163	0.81 (0.60–1.09)	0.170
Activities of daily living (DAD)^b^	1.13 (0.97–1.31)	0.124	1.11 (0.95–1.29)	0.204	1.09 (0.94–1.26)	0.278
Medical history						
Smoking present^a^	1.18 (0.89–1.55)	0.250	1.09 (0.82–1.45)	0.541	1.12 (0.85–1.49)	0.419
Hypertension present^a^	1.24 (0.94–1.64)	0.130	1.11 (0.83–1.49)	0.467	1.10 (0.82–1.47)	0.528
Hypercholesterolemia present^a^	0.86 (0.62–1.19)	0.861	0.73 (0.52–1.01)	0.059	0.75 (0.54–1.05)	0.091
Diabetes mellitus present^a^	0.72 (0.43–1.22)	0.228	0.62 (0.37–1.06)	0.079	0.65 (0.40–1.05)	0.108
Cardiovascular disease present^a^	1.35 (0.99–1.84)	0.060	1.07 (0.77–1.48)	0.700	1.09 (0.78–1.51)	0.625
No. of medications	1.17 (1.03–1.33)	0.017	1.08 (0.95–1.24)	0.251	1.11 (0.97–1.28)	0.125

*Abbreviations: APOE* Apolipoprotein E, *DAD* Disability Assessment for Dementia, *MMSE* Mini Mental State Examination

Data are presented as HR (95% CI) using pooled data of 15 imputations per SD increase for continuous variables or for the presence of the dichotomous variable for mortality

^a^ Dichotomous variable

^b^ Because a lower score indicates worse performance, these scores were inverted

**Table 4:** Cox proportional hazards models used to evaluate influence of cognitive performance, magnetic resonance imaging, and cerebrospinal fluid at baseline on survival

	Model 1 unadjusted		Model 2 adjusted for age and sex		Model 3: model 2 plus MMSE and duration of complaints	
	HR (95% CI)	*p* Value	HR (95% CI)	*p* Value	HR (95% CI)	*p* Value
Cognitive tests						
MMSE^a^	1.11 (0.97–1.28)	0.131	1.23 (1.07–1.42)	0.005	1.25 (1.08–1.44)	0.003
Digit span forward^a^	1.07 (0.93–1.23)	0.362	1.10 (0.95–1.26)	0.207	1.03 (0.89–1.20)	0.651
Digit span backward^a^	1.21 (1.05–1.40)	0.008	1.31 (1.13–1.52)	0.000	1.24 (1.06–1.46)	0.009
VAT naming^a^	1.15 (1.01–1.31)	0.037	1.14 (1.01–1.30)	0.042	1.11 (0.97–1.27)	0.136
VAT memory^a^	1.01 (0.88–1.15)	0.937	1.07 (0.93–1.23)	0.360	1.02 (0.88–1.19)	0.790
TMT-A	1.21 (1.07–1.37)	0.003	1.29 (1.14–1.47)	0.000	1.23 (1.08–1.41)	0.003
TMT-B	1.19 (1.05–1.35)	0.006	1.28 (1.13–1.45)	0.000	1.21 (1.06–1.40)	0.005
RAVLT, immediate recall^a^	1.23 (1.06–1.43)	0.008	1.19 (1.02–1.38)	0.025	1.11 (0.95–1.30)	0.193
RAVLT, delayed recall^a^	0.96 (0.84–1.10)	0.533	0.96 (0.83–1.10)	0.507	0.90 (0.78–1.04)	0.154
Category fluency^a^	1.17 (1.02–1.37)	0.045	1.17 (1.01–1.36)	0.041	1.10 (0.94–1.29)	0.243
MRI						
MTA	1.26 (1.10–1.44)	0.001	1.18 (1.02–1.37)	0.030	1.15 (0.98–1.34)	0.081
PA	1.12 (0.97–1.30)	0.113	1.10 (0.95–1.28)	0.192	1.12 (0.96–1.29)	0.143
GCA	1.21 (1.05–1.40)	0.008	1.18 (1.01–1.37)	0.037	1.17 (1.00–1.36)	0.044
WMH	1.16 (1.01–1.33)	0.041	1.07 (0.92–1.25)	0.364	1.05 (0.90–1.22)	0.518
Lacunes present^b^	1.15 (0.77–1.71)	0.505	1.10 (0.73–1.66)	0.634	1.17 (0.76–1.79)	0.485
Microbleed categories						
Microbleeds, 1–2	0.82 (0.49–1.37)	0.450	0.72 (0.43–1.19)	0.195	0.69 (0.42–1.16)	0.163
Microbleeds, ≥ 3	1.09 (0.80–1.47)	0.598	1.03 (0.76–1.40)	0.840	1.01 (0.74–1.37)	0.956
Infarcts present^b^	1.11 (0.63–2.00)	0.710	1.15 (0.64–2.05)	0.641	1.11 (0.60–2.05)	0.727
CSF						
Aβ_42_^a^	0.98 (0.84–1.14)	0.765	1.02 (0.87–1.18)	0.850	0.99 (0.86–1.16)	0.943
tau	1.05 (0.91–1.22)	0.504	1.09 (0.94–1.27)	0.275	1.07 (0.92–1.26)	0.369
p-tau	1.06 (0.92–1.23)	0.426	1.09 (0.94–1.26)	0.242	1.08 (0.93–1.26)	0.316

*Abbreviations: MMSE* Mini Mental State Examination, *VAT* Visual Association Test, *TMT* Trail Making Test, *RAVLT* Rey Auditory Verbal Learning Task, *MRI* Magnetic resonance imaging, *MTA* Medial temporal lobe atrophy, *PA* Parietal atrophy, *GCA* Global cortical atrophy, *WMH* White matter hyperintensities score 3, *CSF* Cerebrospinal fluid, *Aβ*_*42*_ β-Amyloid 1–42, *p-tau* Tau phosphorylated at threonine 181

Data are presented as HR (95% CI) using pooled data of 15 imputations per SD increase for continuous variables or for the presence of the dichotomous variable for mortality

^a^ Because a lower score indicates worse performance, these scores were inverted

^b^ Dichotomous variable

**Fig. 1:**
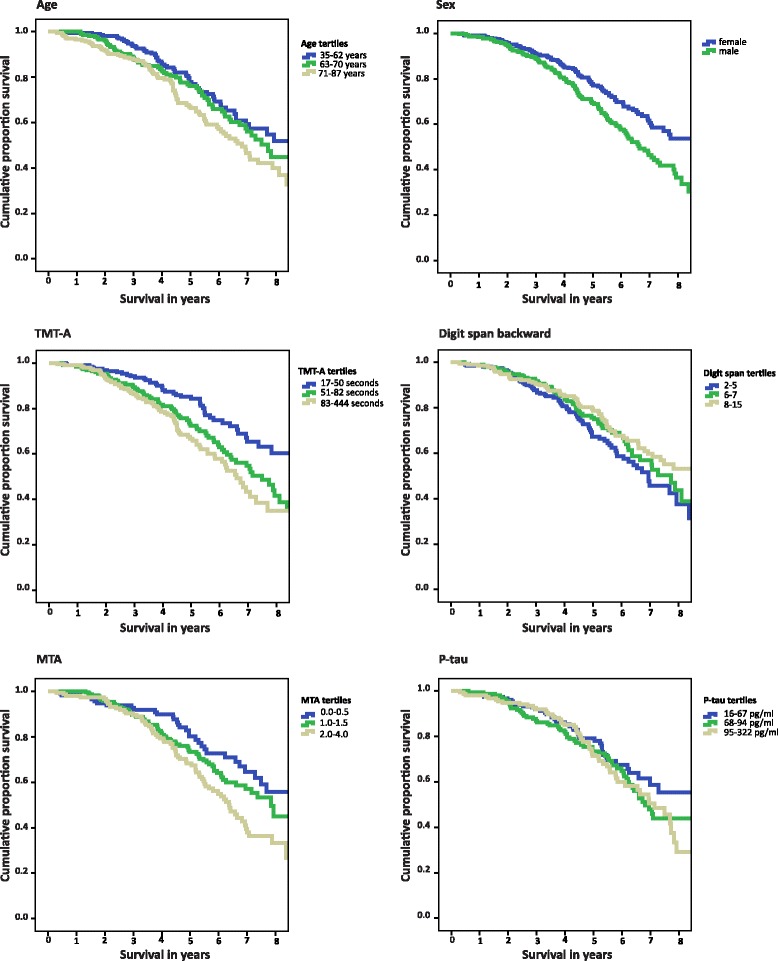
Kaplan-Meier curve, according to variables from forward selection model: age, sex, TMT-A, digit span backward, MTA, and p-tau (all except sex stratified in tertiles). Legend: Note: digit span backward: range 0-21, TMT: trail making test (no range), MTA: medial temporal lobe atrophy ranging 0-4 (average score of left and right side), p-tau: tau phosphorylated at threonine 181. Survival curves were calculated using raw data, without imputation

Next, we aimed to identify the optimal combination of determinants in a multivariate model. With use of forward selection, the model included age (HR 1.31, 95% CI 1.12–1.54, *p* = 0.001), male sex (HR 1.67, 95% CI 1.26–2.21, *p* = 0.000), digit span backward (HR 1.22, 95% CI 1.03–1.43, *p* = 0.018), TMT-A (HR 1.22, 95% CI 1.06–1.41, *p* = 0.005), MTA (HR 1.18, 95% CI 1.01–1.38, *p* = 0.038), and CSF p-tau (HR 1.15, 95% CI 1.00–1.32, *p* = 0.058). Survival curves for these variables are shown in Fig. 1. Of note, because < 50% of our subjects had died, median survival time can only be estimated from these curves.

## Discussion

Our main finding is that despite their relatively young age, roughly one of three patients with AD had died with a mean of 5 years after diagnosis. Predisposing factors for an increased risk of mortality were older age, male sex, more severe executive dysfunction, presence of MTA, and higher p-tau in CSF, indicative of more severe AD pathology. By contrast, duration of complaints, level of activities of daily living, *APOE* ε4 status, and comorbidity were not related to mortality.

In our relatively young population (average age 67 ± 8 years) derived from a tertiary memory clinic cohort with mild to moderate dementia (all with MMSE scores > 16; average MMSE score 22), 35% of the patients had died within 5 years after receiving their baseline diagnosis. This mortality rate is considerably higher than that of the general Dutch population [19]. Previous studies described slightly higher mortality rates, but most studies included patients older than 75 years of age or with severe dementia with MMSE scores < 20 [38–40]. Only a few studies have been focused on mortality in young patients with AD or less affected patients, showing comparable mortality rates [38, 41–43]. Extending these former studies, we evaluated not only comorbidity but also focused on disease-specific markers as determinants of mortality.

In addition to male sex and older age, both of which are known determinants of mortality in AD and in the general population, we found executive dysfunction, MTA, and higher p-tau in CSF, all reflecting more severe disease, to be determinants of mortality. Self-reported duration of complaints was not associated with mortality, indicating that the patients who died had more aggressive rather than more advanced disease. In line with this notion, there was hardly any difference at baseline in the severity of cognitive impairment between those who died within the study period and those who remained alive. Previous studies focused on neuropsychology have shown mainly an association with mortality when assessing decline over time but not at baseline [5, 38, 39]. In our study, we consistently found tests in the executive domain and, to a lesser degree, memory as determinants of mortality. A potential explanation for this finding is that subjects with executive dysfunction are at greater risk of dependency, increasing the risk of complications. Also, the executive domain seems to be a mediator for other cognitive domains, whereas tests for delayed recall were already at floor level in many patients [44]. This latter finding could explain why tests for delayed recall showed no association with survival.

To our knowledge in only two other studies have researchers assessed associations of MRI atrophy markers with mortality, with findings that global atrophy, but not MTA, was associated with mortality in dementia [12, 18]. An association of MTA and mortality in AD was found in a study conducted with computed tomographic scans [45]. In our univariate models, we found more severe MTA and global atrophy associated with increased risk of death; in the multivariate forward selection model, GCA was not included. Atrophy is seen on MRI scans as a marker of downstream neuronal degeneration [25]. In this study, other markers of neurodegeneration, such as p-tau in CSF, were also included in our multivariate forward selection model, which confirms the results of the few studies addressing CSF and mortality in AD [16, 18]. The effect of WMH seems attributable largely to age, because the prognostic value disappeared in the adjusted models. This is different from what has been found before and could potentially be explained by the relatively young age of our sample [12].

In line with previous studies, male sex was associated with higher mortality in AD [3]. It has been suggested that women present earlier in their disease course owing to more easily noticed impairment in household tasks, and hence they have a longer survival time [3]. Also, women more often lived alone and were more frequently widowed, leading to impairment being noticed earlier. We did not find an association with level of activities of daily living (as measured by the DAD). We believe this is possibly most relevant in more advanced disease stages and not in our cohort, where activities of daily living were only mildly impaired in most patients [41, 46]. Finally, and contrary to our expectations, we could not confirm smoking, comorbidity, or number of medications as predisposing factors for an increased risk of mortality. Previous studies have shown an association of cardiovascular risk factors with mortality, but these studies were focused mostly on older populations that are by definition at higher risk of both cardiovascular disease and mortality [11, 13–15, 46]. Also, a higher level of comorbidity has previously been shown to relate to survival time [14, 15], but again in older populations; in our present study, we used number of medications as a proxy of level of comorbidity and found no association [13]. Our study shows that the AD process itself, as reflected by neuropsychology as well as MRI and CSF biomarkers, has prognostic value in terms of mortality as well. This fits with the observation that patients with AD have higher rates of mortality than the general population and that AD is the swiftest growing cause of death in the Western world [2, 19].

Limitations of the present study are that our population was derived from a tertiary memory clinic, which hampers the generalizability of the results. However, the added value of our study is its focus on younger patients, for whom a paucity of data exists. We studied a broad range of determinants in patients with a relatively long follow-up duration. In addition, we included only patients with MMSE scores ≥ 16 to prevent cognitive testing from being at floor level. Of note, even in our young, mild to moderately impaired cohort of patients with AD, mortality was high. Another limitation might be the mean follow-up duration of 5.3 ± 1.8 years for the patients who remained alive, implying that these patients might have died shortly after this period. Nonetheless, all patients had a minimum follow-up of 2 years. Finally, we had information on medication use only at baseline and thus had no information on the prescription of cholinesterase inhibitors after the diagnosis AD. This could be a limitation because some studies have shown that cholinesterase inhibitors can increase survival, whereas others have shown no such effect or only in older patients [41, 43, 47]. Furthermore, we were not able to look at the relationship between use of antipsychotics and mortality [48, 49], because only a very small proportion of our subjects used these medications. However, use of antipsychotics is likely to occur later in the course of the disease. Among the strengths of the present study is our harmonized diagnostic protocol according to which all patients were assessed, because all patients were selected from the same memory clinic and received the same diagnostic workup and similar treatment and management.

## Conclusions

Our results have important clinical implications. We found that AD-related factors, rather than comorbidity or duration of complaints, were associated with increased mortality in our relatively young cohort. This knowledge enables timely dialogue on prognosis, even in young patients who are otherwise healthy.

## Additional file

Additional file 1: Table S1. Cox proportional hazard models using nonimputed data; influence of baseline characteristics and medical history on survival status. **Table S2.** Cox proportional hazard models based on nonimputed data; influence of CSF and MRI on survival status. (DOCX 26 kb)

## Abbreviations

Aβ_42_: β-Amyloid 1–42
AD: Alzheimer’s disease
*APOE*: Apolipoprotein E
CSF: Cerebrospinal fluid
DAD: Disability Assessment for Dementia
FLAIR: Fluid-attenuated inversion recovery
GCA: Global cortical atrophy
MMSE: Mini Mental State Examination
MRI: Magnetic resonance imaging
MTA: Medial temporal lobe atrophy
PA: Parietal atrophy
p-tau: Tau phosphorylated at threonine 181
RAVLT: Rey Auditory Verbal Learning Task
TMT-A: Trail Making Test A
TMT-B: Trail Making Test B
VAT: Visual Association Test
WMH: White matter hyperintensities
