# Supplementary material for: Disease-related determinants are associated with mortality in dementia due to Alzheimer’s disease
Source: Alzheimers Res Ther. 2018 Feb 20;10:23. doi: 10.1186/s13195-018-0348-0 (PMC5819199; doi:10.1186/s13195-018-0348-0)
Supplement: Supplementary file 1 — Cox proportional hazard models using nonimputed data; influence of baseline characteristics and medical history on survival status. Table S2. Cox proportional hazard models based on nonimputed data; influence of CSF and MRI on survival status. (DOCX 26 kb) [file 13195_2018_348_MOESM1_ESM.docx]

**Additional file 1**

**Table S1** Cox proportional hazard models, using non-imputed data; influence of baseline characteristics and medical history on survival status.

|  |  |  | **Model 1 *unadjusted*** | | **Model 2 *adjusted for  age and sex*** | | **Model 3 *model 2 plus MMSE  and duration of complaints*** | |
| --- | --- | --- | --- | --- | --- | --- | --- | --- |
|  |  |  | **HR (95% CI)** | ***p-value*** | **HR (95% CI)** | ***p-value*** | **HR (95% CI)** | ***p-value*** |
| ***Demographics*** | **Gender, male^a^** |  | 1.57(1.20-2.10)) | .001 | 1.61(1.22-2.11) | .001 | 1.76(1.33-2.33) | .000 |
|  | **Age** |  | 1.27(1.11-1.46) | .001 | 1.29(1.11-1.48) | .000 | 1.34(1.16-1.55) | .000 |
|  | **Years of education** |  | 0.99(0.86-1.1.3) | .844 | 0.97(0.84-1.11) | .636 | 1.03(0.90-1.19) | .671 |
|  | **Years of complaints** |  | 0.88(0.75-1.20) | .088 | 0.88(0.76-1.02) | .089 | 0.86(0.74-1.00) | .050 |
|  | **APOE e4 carrier ^a^** |  | 0.79(0.59-1.06) | .109 | 0.81(0.60-1.09) | .168 | 0.81(0.60-109) | .166 |
|  | **Activities of daily living (DAD) ^b^** |  | 1.05(0.86-1.29) | .623 | 1.04(0.85-1.28) | .690 | 1.06(0.86-1.31) | .577 |
| ***Medical history of*** | **Smoking present^a^** |  | 1.18(0.89-1.55) | .249 | 1.10(0.83-1.46) | .529 | 1.12(0.84-1.49) | .440 |
|  | **Hypertension present^a^** |  | 1.24(0.94-1.65) | .130 | 1.11(0.83-1.49) | .467 | 1.08(0.81-1.45) | .608 |
|  | **Hypercholesterolemia present^a^** |  | 0.86(0.62-1.19) | .369 | 0.73(0.52-1.01) | .059 | 0.74(0.53-1.03) | .074 |
|  | **Diabetes mellitus present^a^** |  | 0.72(0.43-1.22) | .228 | 0.62(0.37-1.06) | .079 | 0.64(0.37-1.08) | .095 |
|  | **Cardiovascular disease present^a^** |  | 1.35(0.99-1.84) | .060 | 1.07(0.77-1.48) | .700 | 1.07(0.77-1.49) | .686 |
|  | **Number of medications** |  | 1.17(1.03-1.33) | .017 | 1.08(0.95-1.24) | .251 | 1.10(0.96-1.27) | .167 |

Note: DAD: disability assessment of dementia. Data are presented as hazard ratio (HR) (95% CI) using non-imputed data, per standard deviation increase for continuous variables or for the presence of the dichotomous variable (^a^). ^b^ since a lower score indicates a worse performance, this score was inverted

**Table S2** Cox proportional hazard models based on non-imputed data; influence of CSF and MRI on survival status.

|  |  |  | **Model 1 *unadjusted*** | | **Model 2 *adjusted for  age and sex*** | | **Model 3 *model 2 plus MMSE  and duration of complaints*** | |
| --- | --- | --- | --- | --- | --- | --- | --- | --- |
|  |  |  | **HR (95% CI)** | ***p-value*** | **HR (95% CI)** | ***p-value*** | **HR (95% CI)** | ***p-value*** |
| ***Neuropsychology*** | **MMSE^a^** |  | 1.11(0.97-1.28) | .131 | 1.23(1.07-1.42) | .005 | 1.26(1.09-1.45) | .002 |
|  | **Digit span forward^a^** |  | 1.07(0.92-1.22) | .380 | 1.09(0.95-1.26) | .231 | 1.03(0.89-1.20) | .672 |
|  | **Digit span backward^a^** |  | 1.22(1.05-1.41) | .008 | 1.31(1.12-1.52) | .001 | 1.24(1.05-1.46) | .010 |
|  | **VAT naming^a^** |  | 1.15(1.01-1.30) | .034 | 1.14(1.00-1.29) | .045 | 1.11(0.97-1.26) | .122 |
|  | **VAT memory^a^** |  | 1.01(0.87-1.16) | .915 | 1.07(0.92-1.24) | .370 | 1.04(0.88-1.22) | .647 |
|  | **TMT-A** |  | 1.21(1.06-1.37) | .004 | 1.29(1.13-1.47) | .000 | 1.25(1.09-1.433) | .002 |
|  | **TMT-B** |  | 1.18(1.03-1.34) | .014 | 1.26(1.10-1.44) | .001 | 1.22(1.06-1.40) | .006 |
|  | **RAVLT, immediate recall^a^** |  | 1.20(1.02-1.41) | .025 | 1.16(0.99-1.36) | .072 | 1.08(0.91-1.28) | .385 |
|  | **RAVLT, delayed recall^a^** |  | 0.96(0.83-1.12) | .603 | 0.96(0.83-1.11) | .574 | 0.90(0.77-1.06) | .203 |
|  | **Category fluency^a^** |  | 1.14(0.97-1.33) | .113 | 1.14(0.98-1.34) | .092 | 1.09(0.93-1.28) | .299 |
| ***MRI*** | **MTA** |  | 1.28(1.11-1.47) | .001 | 1.21 (1.03-1.40) | .017 | 1.17(1.00-1.37) | .054 |
|  | **PA** |  | 1.18(1.01-1.37) | .039 | 1.16 (0.99-1.35) | .066 | 1.17(0.99-1.37) | .056 |
|  | **GCA** |  | 1.23(1.06-1.43) | .006 | 1.20(1.03-1.40) | .022 | 1.19(1.02-1.39) | .032 |
|  | **WMH** |  | 1.17(1.01-1.34) | .033 | 1.08(0.93-1.26) | .322 | 1.07(0.92-1.25) | .387 |
|  | **Lacunes present^b^** |  | 1.34(0.80-2.25) | .260 | 1.21(0.72-2.03) | .479 | 1.33(0.79-2.25) | .283 |
|  | **Microbleeds, categories** |  |  |  |  |  |  |  |
|  | **Microbleeds, 1-2** |  | 0.82(0.49-1.36) | .439 | 0.70(0.42-1.17) | .172 | 0.68(0.41-1.15) | .150 |
|  | **Microbleeds, ≥3** |  | 1.71(1.08-2.70) | .022 | 1.53(0.96-2.44) | .072 | 1.49(0.94-2.38) | .091 |
|  | **Infarcts present^b^** |  | 1.72(0.64-4.65) | .284 | 1.89(0.70-5.12) | .210 | 1.84(0.68-4.99) | .232 |
| ***CSF*** | **AB42^a^** |  | 1.01 (0.86-1.20) | .866 | 1.03(0.88-1.21) | .697 | 1.01(0.86-1.18) | .897 |
|  | **t-tau** |  | 1.13(0.99-1.29) | .074 | 1.17 (1.03-1.34) | .017 | 1.17(1.02-1.34) | .025 |
|  | **p-tau** |  | 1.14(0.99-1.31) | .077 | 1.16(1.01-1.33) | .035 | 1.15(1.00-1.32) | .049 |

Note: MMSE: mini mental state examination VAT: visual association test, TMT: trail making test, RAVLT: Rey auditory verbal learning task, MTA: medial temporal lobe atrophy, PA: patrophy, GCA: global cortical atrophy, WMH: white matter hyperintensities 3, AB42: beta amyloid 1-42, p-tau: tau phosphorylated at threonine 181.
^a^: since a lower score indicates a worse performance, these scores were inverted. Data are presented as hazard ratio (HR) (95% CI) using non-imputed data, per standard deviation increase for continuous variables or for the presence of the dichotomous variable (^b^), for mortality.
